# Unveiling the Potential of Algal Extracts as Promising Antibacterial and Antibiofilm Agents against Multidrug-Resistant *Pseudomonas aeruginosa*: In Vitro and In Silico Studies including Molecular Docking

**DOI:** 10.3390/plants12183324

**Published:** 2023-09-20

**Authors:** Shimaa El-Sapagh, Rania El-Shenody, Leonel Pereira, Mostafa Elshobary

**Affiliations:** 1 Department of Botany and Microbiology, Faculty of Science, Tanta University, Tanta 31527, Egypt; shaymaa_elsabagh@science.tanta.edu.eg (S.E.-S.); rania.abdelsamad@science.tanta.edu.eg (R.E.-S.); 2 Department of Life Sciences, University of Coimbra, MARE-Marine and Environmental Sciences Centre/ARNET-Aquatic Research Network, 3000-456 Coimbra, Portugal; leonel.pereira@uc.pt

**Keywords:** algal extract, phytochemical composition, *Pseudomonas aeruginosa*, antimicrobial, antibiofilm, ADME

## Abstract

Multidrug-resistant *Pseudomonas aeruginosa* poses a global challenge due to its virulence and biofilm-forming ability, leading to persistent infections. This study had a dual focus: first, it aimed to investigate the biofilm activity and antibiotic resistance profiles of Pseudomonas aeruginosa isolates obtained from a fish-rearing farm. Second, it explored the potential of algal extracts as effective antibacterial and antibiofilm agents. The study analyzed 23 isolates of *P. aeruginosa* from the farm, assessing antibiotic resistance and biofilm formation. The antimicrobial and antibiofilm activities of two algal extracts, *Arthrospira platensis* (cyanobacteria) acetone extract (AAE) and *Polysiphonia scopulorum* (Rhodophyta) methanol extract (PME), were tested individually and combined (COE). The effects on biofilm-related gene expression were examined. AAE, PME, and COE were evaluated for antimicrobial and antibiofilm properties. Biofilm-related gene expression was measured and the extracts were analyzed for physicochemical properties and toxicity. Most *P. aeruginosa* isolates (86.9%) were antibiotic-resistant and formed biofilms. AAE, PME, and COE displayed promising antibacterial and antibiofilm effects, with COE being particularly effective. COE reduced a key biofilm-related gene expression. The fatty acid content (56% in AAE and 34% in PME) correlated with the effects. Specific compounds, such as phytol, bromophenol, and dihydroxy benzaldehyde, contributed to the activities. The extracts showed favorable characteristics and interactions with FabZ protein amino acids. This study suggests the potential of algal extracts as antibacterial and antibiofilm agents against drug-resistant infections. Further exploration in clinical applications is warranted.

## 1. Introduction

The widespread use of antibiotics has brought about tremendous advancements in healthcare, saving countless lives. However, the overuse and misuse of antibiotics have caused the rapid emergence of multidrug-resistant strains of bacteria (MDR), posing a significant challenge to global healthcare systems [1]. This rise in multidrug-resistant bacteria has raised concerns about a potential “post-antibiotic” era, as highlighted by the World Health Organization [2].

*Pseudomonas aeruginosa* has been identified as one of the most critical pathogens isolated from a community in Egypt and around the world. *P. aeruginosa*, a pathogen commonly found in aquaculture farms, seriously threatens fish health, food safety, and human health. This bacterium is associated with various diseases in fish, including ulcerative syndrome, hemorrhagic septicemia, gill necrosis, and abdominal distension [3,4]. Moreover, it can infect humans through water or food consumption, particularly affecting individuals with compromised immune systems [4,5]. *P. aeruginosa,* a pathogenic bacterium, capitalizes on the rapid development of antibiotic resistance in many of its isolates, as well as a variety of cell surface and secreted virulence factors. These attributes enhance its ability to invade host tissues, resulting in weakened immune systems and an increased pathogenicity. *P. aeruginosa* is among the top multidrug-resistant bacteria (MDR) the WHO has designated as a crucial priority in searching for new therapeutic tactics. *P. aeruginosa’s* resistance to most conventional antibiotics is due to its phenotypic and genotypic characteristics [6]. It has evolved several resistance mechanisms, including the reduced permeability of its outer membrane, efflux pumps to expel antibiotics, and the production of enzymes that inactivate them. *P. aeruginosa* can develop resistance through either the horizontal transfer of resistance genes or mutational changes. Adaptive resistance in *P. aeruginosa* is associated with the formation of biofilms [7].

This versatile Gram-negative bacterium has a diverse metabolic capacity, enabling it to adapt and form biofilms on various biotic and abiotic surfaces [8]. It encloses itself in a protective extracellular polysaccharide matrix that contributes to its high antibiotic tolerance and resistance to the host’s immune system. This matrix allows the bacterium to adhere and colonize effectively, leading to persistent infections. The biofilm’s protective barrier hinders antimicrobial penetration and can also lead to the development of complex drug resistance mechanisms. Additionally, biofilms can modify or inactivate antimicrobial enzymes. However, the sensitivity of *P. aeruginosa* to antibiotics could be restored if the bacteria were to lose the protective biofilm structure [9].

*P. aeruginosa* biofilms consist of three vital exopolysaccharides: alginate, polysaccharide synthesis locus (Psl), and pellicle exopolysaccharides (Pel), which initiate biofilm formation and provide structure [10]. These exopolysaccharides provide bacterial tolerance against antimicrobials and host immunity [11,12]. Bacterial biofilms, including *P. aeruginosa*, contribute to chronic infections, posing risks to immunocompromised individuals and those with cystic fibrosis [8,13]. *P. aeruginosa* biofilms resist antibiotics and host defenses, leading to treatment challenges and impaired immunity [8,13].

For all the previously mentioned reasons, and standard antibiotics finding it challenging to completely remove the biofilms created within the body, developing new therapeutic approaches to combat biofilm-associated *P. aeruginosa* infections is imperative.

In this regard, the search for novel bioactive agents, including biofilm inhibitors and natural compounds, could be highly beneficial [14]. Aquatic organisms, in particular, have a remarkable ability to withstand severe environmental conditions and defend themselves against bacterial invasions, indicating that they may contain a wealth of bioactive compounds [15]. Algae, traditionally used for nutrition and medicine, are a valuable source of bioactive compounds with diverse biological activities, including antitumor, antimicrobial, anti-inflammatory, antiviral, and neuroprotective effects, proven in laboratory and animal studies [16,17,18]. The abundance of algal resources and the diverse chemical compositions of algae highlight their enormous potential for industrial applications [17,19,20]. Several studies have demonstrated that macroalgae and cyanobacteria produce biogenic compounds that possess antimicrobial activity, effectively targeting pathogenic bacteria. These compounds can disrupt the biofilm matrix and eliminate biofilms while preserving other organisms within the ecosystem without causing harm [21].

This study comprehensively analyzes antibiotic resistance and biofilm formation in *P. aeruginosa isolates* from a fish farm, while also exploring the potential of *Arthrospira platensis* (cyanobacteria) and *Polysiphonia scopulorum* (Rhodophyta) extracts as a potential solution to combatting these drug-resistant strains. This dual approach reflects the complexity of addressing antibiotic resistance issues by considering both the problem (resistant strains) and potential solutions (algal extracts). These algae species, known for their diverse bioactivities, have not been extensively studied for their antibiofilm effects. The research examines the impact of algal extracts on bacterial biofilm formation, both at the molecular level and through a microscopic analysis. Furthermore, the study utilizes in silico methods, including ADME prediction and docking studies, to investigate the pharmacokinetics of phycocomponents in algal extracts.

## 2. Results

### 2.1. Isolation, Quantification, and Characterization of Pseudomonas aeruginosa

In this study, 104 bacterial isolates were isolated from 61 water samples taken from a fish farm in the Abu-Arab district, Riyadh, Kufr El-Sheikh Governorate. Of these, 67 Gram-negative bacterial isolates were recovered and identified using conventional identification tests based on colony morphology, pigment production, and biochemical reactions. Among the isolates, 23 were identified as *Pseudomonas aeruginosa*, as they exhibited growths on a cetrimide agar medium, produced a pyocyanin pigment with an aromatic odor, and were nonglucose, lactose, and *sucrose* fermentative and nonlactose fermentative on triple sugar iron and MacConkey agar plates, respectively (Figure S1).

All 23 *P. aeruginosa* isolates were positive for the citrate utilization of gelatinase, oxidase, and catalase tests, as depicted in Figure S1. Based on these results, it could be concluded that all 23 isolates were *P. aeruginosa*.

### 2.2. Antibiotic Susceptibility Profile of P. aeruginosa Isolates (Disc Diffusion Method)

The majority of the *P. aeruginosa* isolates were found to be highly resistant to piperacillin–tazobactam (TPZ), cephalexin (CN), cefoxitin (FOX), and cefsulodin (CFS) (86.9%), while a relatively low resistance of isolates was recorded for azithromycin (AZM) and pipemidic acid (PI) (65.2%). The lowest rates of resistance were observed for AK (amikacin), ciprofloxacin (CIP), and imipenem (IPM) (13.1%, 4.34%, and 4.34%, respectively) (Table 1). According to the susceptibility testing of 14 different antibiotics, all tested *P. aeruginosa* (86.9%) isolates were MDR (resistant to at least one agent in three or more antimicrobial categories), except for the P2, P7, and P8 isolates. As listed in Table 2, five resistance patterns (I–V) were obtained. Pattern I was categorized into three classes (a, b, and c), and pattern II was categorized into two classes (a and b) using different antibiotics. The *P. aeruginosa* isolate P17 showed resistance to all antibiotics tested (TPZ, CN, FOX, CFS, LE, NOR, AZM, STX, PI, TOB, MEM, AK, IPM, and CIP). Resistance to all the tested antibiotics was defined as PDR. Hence, the *P. aeruginosa* isolate P17 was a PDR isolate, whereas the other isolates were MDR *P. aeruginosa* isolates (Table 2).

### 2.3. Biofilm-Producing Potential of P. aeruginosa Isolates

In this study, a quantitative method (colorimetric microtiter plate assay) was used to estimate the ability of *P. aeruginosa* to form biofilms. According to the optical density value, all isolates were biofilm formers and could be classified as strongly adherent at OD > 0.5, weakly adherent at 0.25 > OD > 0.125, and moderate at 0.5 > OD > 0.25. Out of the 23 strains, four strains (17.39%) showed a strong biofilm-producing ability (BPA), eight strains (34.78%) were moderate producers, and the remaining strains (47.8%) were weak, as shown in (Table 3). Therefore, *P. aeruginosa* shedding in water produced biofilms for its survival. Based on previous results, the isolates P1, P13, and P17 were selected for further study, as they were strong biofilm producers and also highly antibiotic-resistant strains.

### 2.4. The Antibacterial Efficacy of Crude Extracts

**Figure 1:**
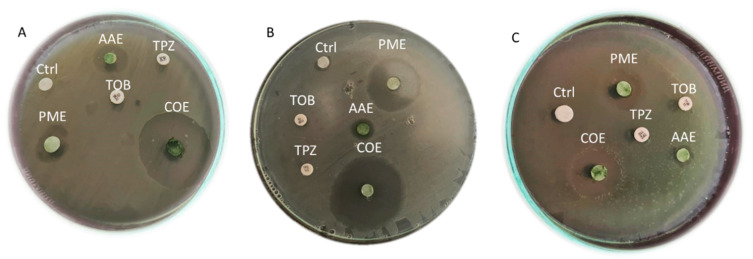
Antimicrobial activity of crude extracts each alone and their combination (at a concentration of 1000µg/ml) against selected *P. aeruginosa* strains (**A**), P1; (**B**), P13 and (**C**). p17. AAE—*A. platensis* acetone extract; PME—*P. scopulorum* methanol extract; COE—combined extract; TOB—Tobramycin; TPZ—Piperacillin +Tazobactam; Ctrl—control.

As shown in Figure 1 and Table 4, the effects of the crude extracts (50 µL/disc at a concentration of 1000 µg/mL) on the tested microbes varied significantly (*p* < 0.001). While the methanol extract of *A. platensis* and the acetone extract of *P. scopulorum* exhibited relatively low antimicrobial activity, it is noteworthy that the *P. scopulorum* methanol extract (PME) and *A. platensis* acetone extract (AAE) demonstrated the most potent antimicrobial activity.

Among the three strains tested, P13 was the most affected by these extracts, while P17 exhibited the least susceptibility. Remarkably, when the AAE and PME were combined (COE), the resulting antimicrobial activity was the most effective, forming clear zones with diameters of 27.66 mm, 36 mm, and 18.33 mm for isolates P1, P13, and P17, respectively. The crude extract activity against the tested pathogens ranked as follows: COE > PME > AAE.

Conversely, the commercial antibiotics tobramycin (10 µg/disc) and piperacillin–tazobactam (TPZ) (75/10 µg/disc) did not exhibit any clear zones of inhibition.

### 2.5. Determination of MIC of Crude Extracts

**Figure 2:**
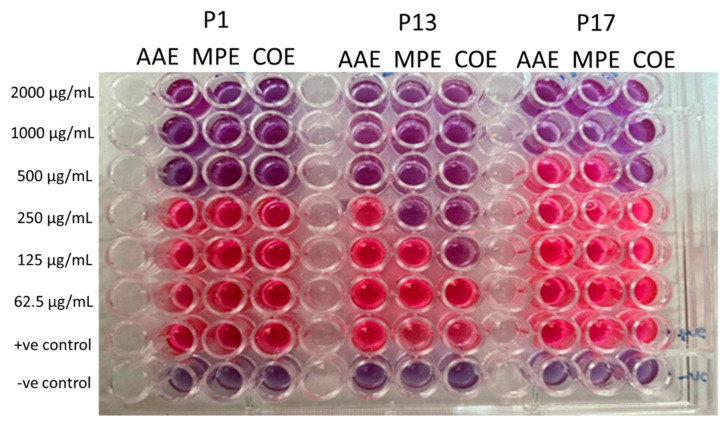
Minimum inhibitory concentration (MIC) values of algal crude extracts determined with resazurin microtiter plates against tested *P. aeruginosa* strains (P1, P13, and P17). AAE, *A. platensis* acetone extract; PME, *P. scopulorum* methanol extract; COE, combined extract. The numbers next to the plate indicate the concentration of algal extract (µg/mL) applied to each column well. +ve control: without the addition of treatment; -ve control: LB broth only.

The ability of different concentrations of the extracts to inhibit the growth of the tested microbial pathogens was investigated to determine the minimum inhibitory concentration (MIC) using the broth microdilution method. This was accomplished by assessing the color change in the resazurin indicator from blue/nonfluorescent to pink/highly fluorescent, indicating microbial metabolism and growth. P1 cells were rendered nonviable after the treatment with 500 µg/mL of all extracts. P13 was inactivated at concentrations of 500, 250, and 125 µg/mL for the AAE, PME, and their combination (COE), respectively (Figure 2). P17 was nonviable at high concentrations of 1000, 1000, and 500 µg/mL for the AAE, PME, and their combination (COE), respectively (Figure 2). This indicated that P13 was more susceptible to the algal extract than P1 or P17, and the combined extract showed the most potent effect.

### 2.6. Determination of the Antibiofilm Activity of Algal Extracts

**Figure 3:**
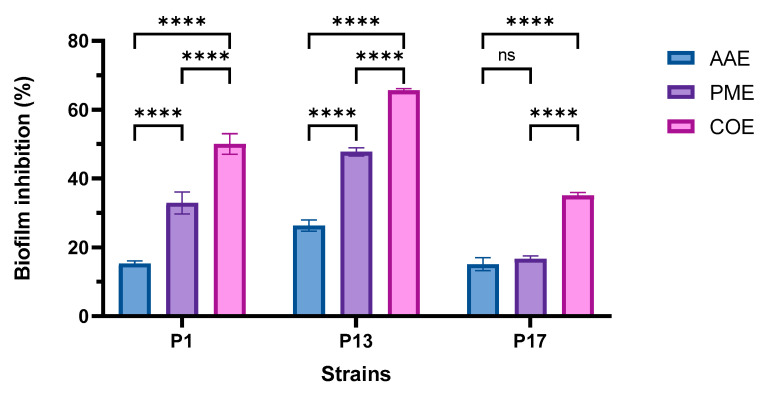
Effect of various algal extracts on the inhibition of biofilm formation using selected *P. aeruginosa* isolates (P1, P13, and P17). Results expressed as mean ± standard deviation of three replicates.**** —very highly significant *p* < 0.0001; ns—not significant *p* > 0.005.

**Figure 4:**
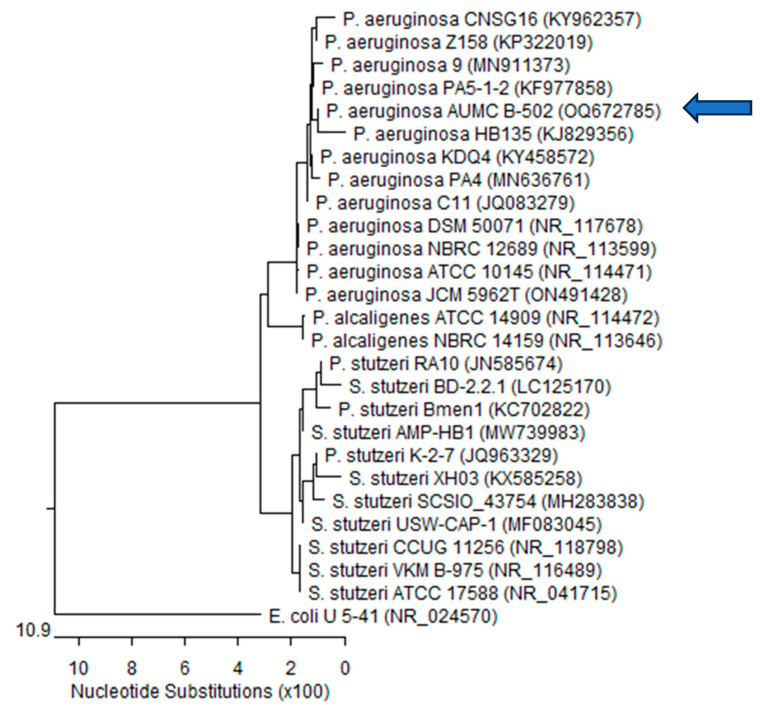
Phylogenetic tree based on 16S rDNA gene sequencing of the bacterial strain *P. aeruginosa* P13 that was named *P. aeruginosa* AUMC-B-502 (GenBank accession no. OQ672785, indicated by the arrow), aligned with closely related sequences of bacterial strains retrieved from GenBank.

As depicted in Figure 3, there was a significant difference in the antibiofilm efficacy of the extracts relative to the three selected isolates. P17 was the most potent strain, with a relatively high resistance to extracts compared to the other tested isolates. Figure 4 shows that the PME had a higher inhibitory effect on the adherence of P1, P13, and P17 than the AAE. Their combination showed the maximum disruptive effect on biofilms formed by the three tested *P. aeruginosa* strains in comparison to each extract alone.

### 2.7. Molecular Identification of MDR P. aeruginosa Strain P13

Only the strong MDR biofilm-producing strain P13, relatively highly sensitive to extracts, was selected for the molecular identification with the 16S rRNA sequencing-based method. The identification and classification of microorganisms up to the species level could be accomplished by sequencing the 16S rRNA genes of the microorganisms. The PCR results showed that the primers successfully amplified the desired gene at the suggested specific length. To identify the isolated strain, the 16S rRNA polymerase chain reaction (PCR) product was purified and sequenced.

The resulting sequences were aligned using BLASTn alignment software (NCBI) against the recognized sequences in GenBank and a phylogenetic tree was constructed using MegAlign (DNA Star) software version 5.05, as shown in Figure 4. The size of the 16S rRNA ribosomal PCR-amplified product was 1402 bp for P13. The 16S rRNA sequencing revealed a 99.86% identity, 100% identity, and 99–100% coverage with several *P. aeruginosa* strains, including the type material *P. aeruginosa* ATCC17588, with GenBank accession no (NR_041715). *Escherichia coli* was included in the tree as an outgroup strain: P.—*Pseudomonas*; E.—*Escherichia*; S.—*Stutzerimonas*. Accordingly, our isolate was named *P. aeruginosa* AUMC B-502, to be deposited in the culture collection of the Botany Department, Faculty of Science, Tanta University, and its sequenced genes were subsequently submitted to GenBank on 21 March 2023 with the accession number OQ672785.

### 2.8. Effect of the Algal Extract on the Biofilm Architecture of P. aeruginosa Using SEM

**Figure 5:**
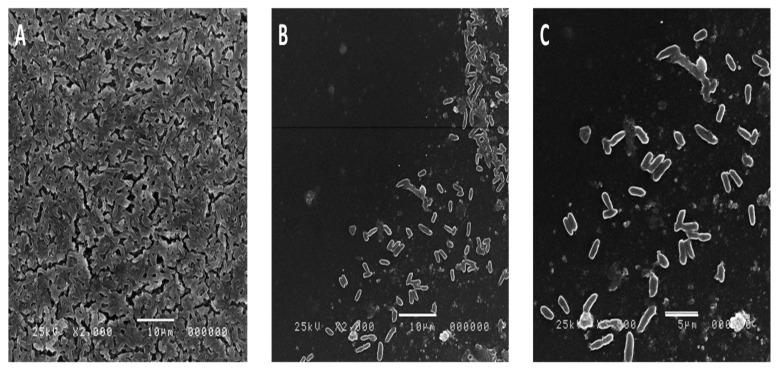
Scanning electron micrographs illustrating the morphological response of Pseudomonas aeruginosa to the combined extract: (**A**) control without treatment; (**B**) treated with the combined extract (2000× magnification); (**C**) treated with the combined extract (3500× magnification).

The biofilm architecture of *P. aeruginosa* P13 with and without the combined algal extract (as the most potent extract) was examined after a 48 h incubation period. Figure 5 represents the micrograph, captured using SEM imaging, of the biofilm produced by the *P. aeruginosa* growth in the presence and absence of the extracts. As shown in Figure 5, the bacterial cells treated with the extracts showed a decrease in the number of cells. The control sample showed a greater number of cells adhering to the surface, whereas the subsequent images showed a reduction in the number of biofilm-forming cells. The control bacteria exhibited multiple layers of bacterial biofilms (Figure 5A). In contrast, the bacterial biofilms treated with the minimum inhibitory concentration (1/2 MIC) displayed a significant decrease in the number of adherent bacteria (Figure 5B,C).

Moreover, a noticeable reduction in biofilm formation was observed. Furthermore, the treated bacterial biofilms exhibited distorted bacterial cells. These observations strongly indicated the potent antibiofilm action of the combined algal extract against *P. aeruginosa*.

### 2.9. Determination of Gene Expression with Quantitative RT-PCR

The *P. aeruginosa* biofilms consisted of three crucial exopolysaccharides: alginate, Psl, and Pel. Among these, alginate is regulated by the AlgACD operon, with the *algD* gene playing a key role in synthesizing the precursor GDP-mannuronic acid and controlling alginate synthesis and the transcription of the Alg proteins [22].

Psl is a mannose-rich polymer that plays a key role in initiating biofilm development and supplies structural integrity to the biofilm. Its synthesis by the Psl operon (pslA to pslO), PslD, located in the periplasm or outer membrane, is crucial for biofilm formation [23]. Pel, another key exopolysaccharide, contains galactosamine and glucosamine, contributing to the biofilm’s integrity [24]. The Pel operon (PelA to PelG) controls Pel synthesis, which is essential for *P. aeruginosa*’s survival in various environments [25]. PelF, a glycosyltransferase in the pel operon, plays a key role in Pel synthesis [26].

These exopolysaccharides create a protective barrier, providing bacterial tolerance against antimicrobials and the immune system [11,12]. P. aeruginosa biofilms, including those formed in our study, have implications for chronic infections and treatment challenges, as they resist host defenses and antibiotics [8,13,27,28].

**Figure 6:**
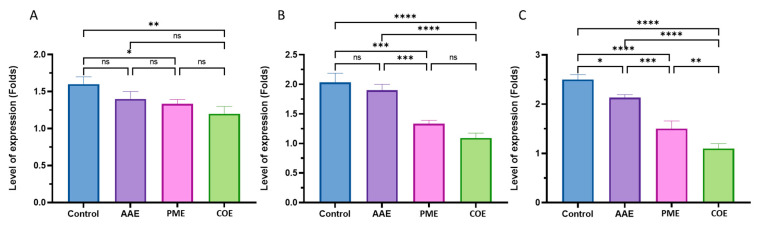
Quantitative real-time PCR (qRT-PCR) analysis of three biofilm-forming genes, namely, (**A**) algD, (**B**) pelF, and (**C**) pslD, under three different algae treatments: *A. platensis* acetone extract (AAE), *P. scopulorum* methanol extract (PME), and their combination (COE). The *y*-axis represents the relative expression levels of the target genes, indicated as relative mRNA abundance. Results expressed as mean ± standard deviation of three replicates. **** —very highly significant *p* < 0.0001; ***— highly significant at *p* < 0.001; **—significant at *p* < 0.01; *—significant at *p* < 0.005; ns—not significant *p* > 0.005.

qRT-PCR was used to determine the expression of the *P. aeruginosa* biofilm genes pelF, pslD, and algD. The P13 isolate was tested in the presence of sub-MIC levels of the extracts extracted (½ × MIC) from the *A. platensis* acetone extract, *P. scopulorum* methanol extract and their combination. P13 was enrolled in this experiment because of its high biofilm intensity and its sensitivity to the algal extract. The results revealed a downregulation effect in the pslD and pelF genes, as well as minor variations in the algD gene. The suppression of these genes with the algal extract confirmed the antibiofilm activity of these extracts on the gene level. algD is responsible for producing D-mannuronic acid and L-guluronic acid, which play an important role in the structural stability and protection of the biofilms. The results of this study demonstrated a statistically significant (*p* = 0.004) downregulation of algD under the combination treatment, with a decrease of 25% from 1.6 in the untreated group to 1.2 in the treated group, while there were no significant differences (*p* = 0.104) between the AAE and control treatment (Figure 6A).

Conversely, the expression patterns of the pelF and pslD genes differed from that of algD, with a significant (*p* < 0.001) downregulation observed in all treatments, except for the AAE treatment, compared to the untreated group. The combination treatment showed the highest gene expression reduction, with a 45% and 56% decrease observed in the pelF and Psl genes, respectively (Figure 6B,C).

### 2.10. Chemical Analysis of the Algal Extract Used in This Study Using GC–MS Chromatograms

It was imperative to define the chemical composition of the algal extracts to find the bioactive constituents that had antimicrobial and antibiofilm activities. The GC–MS chromatograms showed various compounds in the *A. platensis* acetone extract and *P. scopulorum* methanol extract. The names, chemical constituents, molecular weight, molecular formula, and peak area of each component were listed in Table 5 and Table 6. Concerning the acetone extract of *A. platensis,* eight major compounds (>5%) were present as follows: 3,7,11,15-tetramethyl-2-hexadecen-1-ol (phytol) (36.60%), *n*-hexadecanoic acid (palmitic acid) (21.01%), 11,14-eicosadienoic acid, methyl ester (12.68%), trans-13-octadecenoic acid (7.67%), and linoleic acid ethyl ester (7.42%) (Table 5).

The methanol extract of *P. scopulorum* contained five major compounds, each representing more than 5% of the extract’s composition. These compounds and their respective percentages were as follows: palmitic acid (22.74%), bis (3-bromo-4,5-dihydroxybenzyl) ether (13.24%), 3-bromo-4,5-dihydroxybenzaldehyde (10.76%), 3,4-dihydroxybenzaldehyde (9.70%), and Linolenic acid methyl ester (8.69%) (Table 6).

### 2.11. Physicochemical Properties of the Algal Extracts

Physiochemical analyses of the major bioactive compounds in the AAE and PME were analyzed. The analysis in Tables S1 and S2 revealed that the analyzed compounds in the algal extract exhibited optimal property ranges within specific parameters. The molecular weight (MW) of these compounds fell within the range of 200 to 600. The logarithm of the partition coefficient (M log P) ranged from one to five, demonstrating the lipophilicity of the compounds, with the exception of phytol, which had a value of 5.91. The number of hydrogen bond acceptors was between 0 and 4, while the number of hydrogen bond donors ranged from 0 to 5. The topological polar surface area (TPSA) was within the range of <60, with the exception of bis (3-bromo-4,5-dihydroxybenzyl) ether, which had a value of 90.15 Å2. The molecular refractivity (MR) also fell between 40 and 130, reflecting their molecular volume. These findings provided valuable insights into the optimal property ranges of the compounds in the algal extract.

### 2.12. Water Solubility, Pharmacokinetics, Drug Likeness, and Toxicity

Among the identified bioactive compounds in the AAE, the most proportional compounds of the peak area >5% were analyzed, namely, linoleic acid ethyl ester, 3,7,11,15-tetramethyl-2-hexadecen-1-ol, *n*-hexadecanoic acid, 11,14-eicosadienoic acid methyl ester, and trans-13-octadecenoic acid. These compounds had different characteristics and properties (Table S1).

In terms of solubility, linoleic acid ethyl ester was classified as being soluble, according to the ESOL solubility model, and moderately soluble, according to the Silico-it solubility model. Phytol, palmitic acid, methyl 11,14-eicosadienoate, and elaidic acid were all moderately soluble according to both models, except for methyl 11,14-eicosadienoate, which was classified as being poorly soluble in the Silico-it model.

In pharmacokinetics, linoleic acid ethyl ester was not a CYP1A2 inhibitor, but inhibited CYP3A4. *N*-hexadecanoic acid, 11,14-eicosadienoic acid methyl ester, and trans-13-octadecenoic acid were CYP1A2 inhibitors, while 3,7,11,15-tetramethyl-2-hexadecen-1-ol was not a substrate for any of the listed enzymes.

The compounds had different log Kp (cm/s) values, which indicated their permeability across biological membranes. Linoleic acid ethyl ester had a log Kp of −4.85, while the other compounds had less negative log Kp values, suggesting lower permeability.

In terms of drug-likeness, linoleic acid ethyl ester had no Lipinski rule violations and a bioavailability score of 0.55. All the other compounds had one Lipinski rule violation and a bioavailability score of 0.55 and 0.85. None of the compounds had any PAIN (pan assay interference compound) alerts, indicating they were not problematic substances.

The major bioactive compounds found in the MPE were extensively examined, including linolenic acid methyl ester, palmitic acid methyl ester, 3-bromo-4,5-dihydroxybenzaldehyde, bis (3-bromo-4,5-dihydroxybenzyl) ether, and 3,4-dihydroxybenzaldehyde. These compounds had different characteristics and properties (Table S2).

Regarding solubility, both the ESOL and Silicos-it solubility models indicated that linolenic acid methyl ester and bis (3-bromo-4,5-dihydroxybenzyl) ether were moderately soluble. *n*-hexadecanoic acid methyl ester was soluble according to both models, while 3-bromo-4,5-dihydroxybenzaldehyde was soluble in the Silicos-it model and highly soluble in the ESOL model. 3,4-dihydroxybenzaldehyde was highly soluble in both models.

In terms of pharmacokinetics, linolenic acid methyl ester and bis (3-bromo-4,5-dihydroxybenzyl) ether inhibited CYP1A2, while *n*-hexadecanoic acid methyl ester and 3-bromo-4,5-dihydroxybenzaldehyde inhibited CYP2C9. 3,4-dihydroxybenzaldehyde inhibited CYP2C19 and inhibited CYP3A4.

The compounds displayed different log Kp (cm/s) values, reflecting their permeability across biological membranes. Bis (3-bromo-4,5-dihydroxybenzyl) ether had the most negative log Kp value (−6.71), suggesting higher permeability. Linolenic acid methyl ester had a log Kp value of −4.02, while the remaining compounds had less negative log Kp values, indicating lower permeability.

Regarding drug-likeness, linolenic acid methyl ester broke one of Lipinski’s rules with a bioavailability score of 0.85, indicating its relative bioavailability. Only 3-bromo-4,5-dihydroxybenzaldehyde and 3,4-dihydroxybenzaldehyde exhibited a PAIN alert, suggesting that they may have potential for promiscuous or nonspecific interactions with biological targets.

The compounds exhibited varying degrees of synthetic accessibility. Linolenic acid methyl ester had the highest score (3.03), indicating a relatively more straightforward synthesis, while the other compounds had scores ranging from 1 to 2.71.

The cytotoxicity and hepatotoxicity were evaluated using the protox_II online server, which predicted whether the isolated molecules were toxic. Based on the predictive results (Tables S1 and S2), none of the identified phycocompounds were expected to present any toxicity problems.

**Figure 7:**
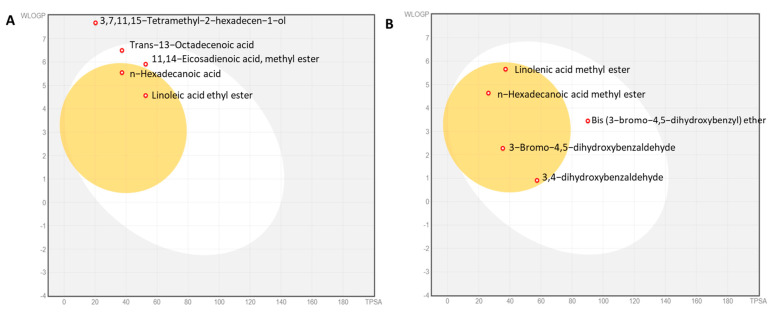
BOILED–Egg model of the dominant phycocompound. (**A**) *A. platensis* acetone extract (AAE) and (**B**) *P. scopulorum* methanol extract (PME).

The BOILED-Egg model provides insights into the absorption and distribution properties of compounds. The white region represents a favorable passive absorption in the gastrointestinal tract, indicating good oral bioavailability. The yellow region, resembling the yolk, suggests a high probability of blood–brain barrier (BBB) penetration, indicating potential central nervous system activity. The blue color indicates a substrate status for P-glycoprotein (PGP+), a drug transporter involved in efflux, while the red color indicates a nonsubstrate status (PGP-). Based on this analysis, the dominant compounds in both the AAE and PME were nonsubstrates of P-glycoprotein (PGP-) and not actively effluxed. Among the top compounds in the AAE, 11,14-eicosadienoic acid methyl ester and trans-13-octadecenoic acid were in the white zone, suggesting good gastrointestinal absorption. Linoleic acid ethyl ester and *n*-hexadecanoic acid were in the yolk region, indicating potential BBB penetration. However, 3,7,11,15-tetramethyl-2-hexadecen-1-ol fell outside the egg, suggesting a limited potential for both gastrointestinal absorption and BBB penetration (Figure 7A). In the PME, all compounds were in the yolk zone, except for bis (3-bromo-4,5-dihydroxybenzyl) ether, which was in the white zone, implying limited BBB penetration (Figure 7B).

### 2.13. Docking Studies

**Figure 8:**
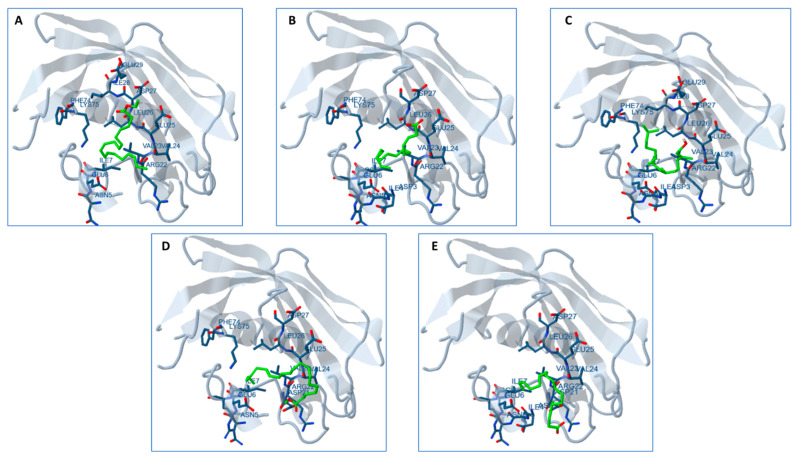

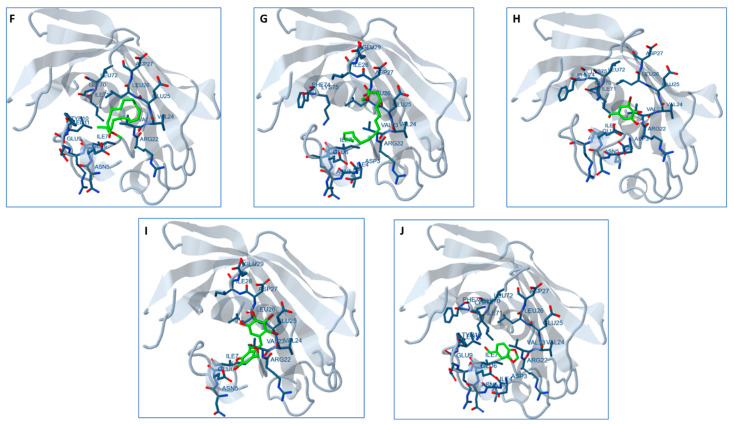
Molecular docking models for phytocompounds of AAE (linoleic acid ethyl ester, (**A**); *n*-hexadecanoic acid, (**B**); 3,7,11,15-tetramethyl-2-hexadecen-1-ol, (**C**); 11,14-eicosadienoic acid, methyl ester, (**D**); trans-13-octadecenoic acid, (**E**)) and PME (linolenic acid methyl ester, (**F**); *n*-hexadecanoic acid methyl ester, (**G**); 3-bromo-4,5-dihydroxybenzaldehyde, (**H**); bis (3-bromo-4,5-dihydroxybenzyl) ether, (**I**); 3,4-dihydroxybenzaldehyde, (**J**).

The molecular docking analysis aimed to assess the interaction of the ten bioactive compounds present in the AAE and PME with the substrate-binding tunnel of the FabZ protein in *P. aeruginosa (*Figure 8*)*. All compounds demonstrated varying degrees of interaction with the target protein. Regarding the binding affinity, compounds with more negative estimated free energy values generally indicates a stronger binding to the target site. For example, “Bis (3-bromo-4,5-dihydroxybenzyl) ether” and “3-Bromo-4,5-dihydroxybenzaldehyde” from the PME, along with “11,14-Eicosadienoic acid, methyl ester” from the AAE, exhibited notably lower free energy values (−4.59, −4.54, and −2.50 kcal/mol, respectively), signifying favorable binding interactions.

The estimated inhibition constant (Ki) further characterized the compounds’ potency as inhibitors. Lower Ki values suggested a higher inhibitory potential. In this context, “Bis (3-bromo-4,5-dihydroxybenzyl) ether,” “3-Bromo-4,5-dihydroxybenzaldehyde” from the PME and “11,14-Eicosadienoic acid, methyl ester” from the AAE displayed lower Ki values of 471.83 µM, 430.74 µM, and 14.71 mM, respectively, indicating a more substantial inhibitory potential compared to the other compounds (Table S3).

There is potential for synergistic effects through combining these extracts, where compounds from both extracts collaboratively enhance the overall activity. The docking results are visually depicted in Figure 8, illustrating the interactions between the bioactive compounds and FabZ. Notably, these compounds displayed binding interactions with ten distinct amino acids within the FabZ protein, including ASP3, VAL23, TYR10, ILE7, ILE28, ILE71, LEU26, LYS75, GLU6, and GLU25. These robust bindings to the FabZ protein suggested their potential to inhibit the enzyme and, consequently, to impede pathogen growth. Given the presence of FabZ in various human pathogens, these phycocompounds could hold promise as potential candidates for the development of broad-spectrum antibiotic drugs.

## 3. Discussion

*P. aeruginosa*, a dangerous pathogen affecting both aquaculture and public health, can cause severe diseases in fish and pose a risk to humans. Our study focused on *Pseudomonas*, a complex genus prevalent in aquaculture systems [3,4]. Exploring new antimicrobial agents from natural sources such as algae is crucial in addressing multidrug-resistant bacteria such as *P. aeruginosa*.

This study aimed to detect and assess *P. aeruginosa* in an Egyptian fish-rearing farm, evaluating its biofilm activity and antibiotic resistance. Among 67 Gram-negative isolates obtained from 61 water samples, 23 were identified as being *P. aeruginosa*. These isolates were tested for antibiotic sensitivity, showing high efficacy against ciprofloxacin, imipenem, and amikacin. These findings aligned with previous research demonstrating the effectiveness of imipenem and amikacin against *P. aeruginosa* [29].

The susceptibility testing revealed a concerning level of resistance among the isolates, particularly to antibiotics such as cephalexin, cefoxitin, cefsulodin, and piperacillin–tazobactam (an antipseudomonal beta-lactam). Alarmingly, 86.9% of the isolates exhibited resistance to these antibiotics. This escalating resistance of *P. aeruginosa* to commonly prescribed antibiotics presents a significant challenge in effectively treating infections caused by this pathogen. Imipenem, piperacillin–tazobactam, cefepime, cefotaxime, and gentamicin, typically used as first-line antibiotics against P. aeruginosa infections, are now facing increased limitations in their efficacy [30]. However, the increasing resistance of *P. aeruginosa* to these drugs presents a major challenge for physicians. This bacterium possesses virulence factors that enable it to evade the host’s immune system and cause damage to host tissues [30]. The development of alternative drugs, such as ciprofloxacin and tobramycin, has been necessary in addressing the resistance issue. According to the current study, our isolates were also resistant to these alternative drugs. Addressing the resistance of *P. aeruginosa* to antibiotics is an urgent concern that requires immediate attention.

In the next phase of our study, we assessed the isolates’ biofilm-forming capabilities, a critical aspect of P. aeruginosa’s pathogenicity. Biofilms enable these bacteria to attach to host surfaces, evade the immune system, and resist treatment, posing significant risks to the host. All the isolates in our study demonstrated biofilm formation using the microtiter plate method with the crystal violet assay. Pseudomonas aeruginosa is well-known for its proficiency in forming biofilms in diverse environments [31,32], aiding its survival and persistence across various conditions. Biofilm phenotypes in our study were categorized as follows: 4 isolates (17.39%) displayed strong adherence, 8 isolates (34.78%) showed moderate adherence, and 11 isolates (47.82%) exhibited weak adherence.

Consistent with findings from [33], our study also demonstrated a noteworthy association between the ability to form biofilms and susceptibility to antibiotics. It was observed that a significantly higher production of biofilms in isolates was multidrug-resistant (MDR) or pandrug-resistant (PAN). The biofilm matrix provides an additional layer of resistance for bacteria. Within biofilm communities, antibiotic resistance can arise through various mechanisms, including the slow or partial penetration of antibiotics into the biofilm and the presence of an altered chemical microenvironment. A subpopulation of microorganisms within the biofilm can also contribute to antibiotic resistance. These findings were compatible with previous investigations in different bacterial species, including *P. aeruginosa* [34,35].

Multiple studies have consistently reported that various algal extracts hold significant potential as a valuable source of novel bioactive molecules with medicinal applications [36,37]. These extracts have demonstrated positive antibacterial activity against diverse strains of pathogens [18,38]. The variance in bioactive compounds can be attributed to factors such as the solvent type, extraction method, and the season during which the algae were collected [39,40]. Our own findings revealed that the methanol extract of *A. platensis* and the acetone extract of *P. scopulorum* exhibited no antimicrobial activity. However, the *A. platensis* acetone extract (AAE) and the *P. scopulorum* methanol extract (MPE) showed strong antimicrobial and antibiofilm activity. This may be attributed to the different bioactive compounds extracted using different solvents that depend on the solubility and polarity of such bioactive compounds. Contrary to previous findings, the *A. platensis* acetone extract (AAE) and the *P. scopulorum* methanol extract (MPE) exhibited significant antimicrobial and antibiofilm activity. This disparity in results may be attributed to the specific bioactive compounds extracted using different solvents, which depend on the solubility and polarity of these compounds. Varying solvents used for extraction can yield different profiles of bioactive compounds, potentially explaining the observed differences in the antimicrobial and antibiofilm efficacy [18,41]. The acetone extract of *Pleurastrum minutum* (formerly *Chlorococcum minutium*) (Chlorophyta) exhibited the highest antimicrobial activity among the tested extracts and demonstrated moderate antioxidant activity. On the other hand, the methanol extract of the same microalga displayed the highest antioxidant activity and moderate antimicrobial activity. These findings highlighted the importance of the choice of solvent during the extraction process, as it plays a crucial role in selectively extracting specific bioactive compounds with varying properties [18].

The observed antibacterial and antibiofilm activity of the algal extracts could be attributed to the presence of bioactive compounds possessing antimicrobial and antibiofilm properties. Algae are known to produce a diverse range of secondary metabolites, including alkaloids, phenolic compounds, terpenes, and polysaccharides, which have demonstrated antimicrobial and antibiofilm activities in previous research [21,37,42]. When different algal extracts were combined, synergistic effects could arise, where the collective action of multiple bioactive compounds enhanced the overall antibacterial efficacy and facilitated biofilm disruption. This promising approach of using combined algal extracts holds potential in effectively combatting persistent infections caused by multidrug-resistant (MDR) and pandrug-resistant (PDR) strains of *P. aeruginosa.* The SEM analysis provided further confirmation that the combined algal extract led to significant distortion and profound morphological changes in the established bacterial biofilms. These findings proved the potential of this combination in preventing and treating bacterial diseases. With further validation and research, this extract could serve as a substitute natural therapeutic drug for eradicating bacterial biofilm infections.

To support our findings, we assessed the gene expression of three crucial genes, namely, algD, pslD, and pelF, in the most resistant strain, P13. These genes play pivotal roles in synthesizing psl, pel, and alginate exopolysaccharides, which are essential for surface attachment, biofilm formation, and the overall stability of the biofilm architecture [32,43]. The algD gene is vital in regulating alginate biosynthesis, including the transcription of alg proteins and the production of GDP-mannuronic acid, a precursor necessary for polymerization and alginate synthesis. pslD contributes significantly to biofilm formation by aiding cell adhesion to surfaces and facilitating cell-to-cell interactions in both nonmucoid and mucoid strains. pslD forms a helical structure during biofilm formation and is regulated through a complex network of factors. Similarly, pel is a critical component of the biofilm matrix in nonmucoid strains, being responsible for the formation of pellicle biofilms [25]. Pel’s presence in the biofilm matrix enhances the structural stability and cohesion, enabling the biofilm to persist and thrive in diverse environments.

In our study, combined algal extract treatments led to a significant downregulation in the algD, pslD, and pelF genes, with their expression decreasing by 25%, 45%, and 56%, respectively, compared to the untreated control. Among individual algal extracts, the acetone extract of A. platensis (AAE) exhibited the lowest effect on gene expression, suggesting a relatively weaker impact on regulating these genes than the other extracts.

These findings underscored the pronounced inhibitory effect of combined algal extract treatments on the expression of algD, pslD, and pelF genes, crucial for biofilm formation and stability. The differing effects of individual algal extracts suggested that the unique composition and bioactive compounds in each extract contributed to variations in their ability to downregulate gene expression. In a related context, extracts from three marine macroalgae (Cladostephus spongiosus—Phaeophyceae; *Codium tomentosum*—Chlorophyta; and *Palisada perforata*—Rhodophyta) inhibited the hyphal growth and biofilm biosynthesis of Candida by downregulating the expression of hyphal-specific genes and reducing the exopolysaccharide layer [44].

To explore the bioactive compounds in the algal extracts, GC–MS was used for both of the bioactive extracts AAE and PME. Both algal extracts were rich in fatty acid content, representing 56% and 34% for the AAE and PME, respectively. Similar to the findings in our study, previous research identified the presence of long-chain aldehydes derived from various fatty acids, including palmitic acid, linoleic acid, oleic acid, and linolenic acid, in both micro- and macroalgae [45,46,47,48,49].

Fatty acids are considered the next generation of antimicrobials and antibiofilm agents [50]. Unsaturated fatty acids have been shown to exhibit significant antibacterial activities against various pathogens, including *Neisseria gonorrhoeae, Pseudomonas aeruginosa, Fusobacterium nucleatum, Neisseria gonorrhoeae*, *Porphyromonas gingivalis,* and *Helicobacter pylori* [30,51,52,53]. Linoleic acid (C18:2) and eicosadienoic acid (C20:2) are a kind of polyunsaturated fatty acid that are present in the AAE. Linoleic acid has antibacterial activity against *Pseudomonas aeruginosa, Staphylococcus aureus,* and *Escherichia coli* [50,54]. Eicosadienoic acid methyl ester, another unsaturated fatty acid present in the AAE, also has antibacterial activity towards Gram-positive bacteria (*Bacillus subtilis, B. cereus,* and *Staphylococcus aureus*) and Gram-negative bacteria (*E. coli, P. aeruginosa*, and *S. typhi*) [55]. Linolenic acid (C18:3) is an unsaturated fatty acid present in the PME; linolenic acid can also enhance the antimicrobial activity of tobramycin against *Pseudomonas aeruginosa* biofilms by interfering with its quorum sensing and reducing its virulence factors [56]. However, linolenic acid may not be able to penetrate the outer membrane of some Gram-negative bacteria, such as *Pseudomonas phaseolicola*, and may require higher doses or liposomal formulations to exert its antibacterial effect [57]. Palmitic acid (C18:0), a saturated acid recorded to exist in the AAE and PME, also showed potent antimicrobial activity against vancomycin-resistant *Enterococcus faecalis* and multidrug-resistant *Staphylococcus epidermidis* [50,58] as well as different oral pathogens [59]. The mechanisms of the action of antibacterial fatty acids are not fully understood, but they may involve the disruption of bacterial membranes, inhibition of enzymes, interference with quorum sensing, and modulation of gene expression [50]. Fatty acids have shown promising potential as next-generation antibacterial agents for the treatment and prevention of bacterial infections, particularly those caused by multidrug-resistant bacteria. Their unique properties and mechanisms of action make them attractive candidates for combating antibiotic resistance [50,60].

The AAE is rich in phytol (3,7,11,15-tetramethyl-2-hexadecen-1-ol), at a concentration of 36.60%. Phytol, a natural compound found in photosynthetic organisms such as algae, has been shown to have inhibitory effects on biofilm formation and the virulence factors of certain bacteria, including *Klebsiella pneumoniae* and *Serratia marcescens* [61,62]. Phytol interferes with the quorum-sensing system of *Klebsiella pneumoniae*, leading to the inhibition of biofilm formation and virulence factors [62].

3-Bromo-4,5-dihydroxybenzaldehyde and bis (3-bromo-4,5-dihydroxybenzyl) ether are natural bromophenols, representing approximately 23% of the PME. These have been previously isolated from marine red macroalgae, such as *Polysiphonia morrowii, Odonthalia corymbifera,* and *Symphyocladia latiuscula* [63,64,65]. 3-Bromo-4,5-dihydroxybenzaldehyde was shown to reduce the production of inflammatory mediators, such as nitric oxide, prostaglandin E2, and cytokines, by inhibiting the activation of a signaling pathway. It was also found that these compounds could suppress the production of reactive oxygen radicals that cause oxidative stress and injury to cells. These bromophenols have also exhibited antiviral activity against the infectious hematopoietic necrosis virus (IHNV) and infectious pancreatic necrosis virus (IPNV), both of which are highly pathogenic viruses affecting fish. A study conducted by Kim et al. [66] demonstrated that bromodichloromethane exhibited an effect against both viruses, whereas bromo-dichloroethane only showed an effect against IPNV. Bromophenol from the red algae *Polyopes lancifolius* showed antimicrobial activity towards *Saccharomyces cerevisiae* and *Bacillus stearothermophilus* (α-glucosidase-producing microorganisms). Bromophenols were reported as inhibitors for DisA synthetase, an enzyme involved in the synthesis of c-di-AMP. This inhibition has been shown to regulate a range of cellular processes in bacteria and archaea, including cell wall synthesis, the stress response, virulence, and sporulation [67]. Inhibiting DisA or other c-di-AMP synthases could be a potential strategy of interfering with bacterial signaling and survival [68].

The compound 3,4-dihydroxybenzaldehyde, which constitutes 9.71% of the MPE in this study, was extracted from the leaves, stems, and roots of the *Trichomanes chinense* fern. It has demonstrated noteworthy antioxidant activity using the DPPH assay. Additionally, it has exhibited antimicrobial properties against various bacteria, including *Vibrio cholerae, Escherichia coli, Staphylococcus aureus*, and *Salmonella thypimurium*.

It is important to note that these studies used bioactive-rich extracts, and the effects observed may have varied depending on different conditions and concentrations. The exact mechanisms through which these bioactive exert their antibacterial effects are not fully understood, but could involve disrupting the bacterial cell membrane, interfering with bacterial signaling pathways, or modulating the host immune response [61,62].

On the other hand, most of the explored bioactive compounds that had antibacterial activity showed antibiofilm activity. For instance, the presence of palmitic acid as the main fatty acid in both algal extracts, with 21.01% for the AAE and 22.75% for the PME, exhibited potent antibiofilm properties. Palmitic acid has demonstrated inhibitory effects on biofilm formation and has been shown to reduce various virulence factors in *Candida tropicalis*, including ergosterol biosynthesis, cell surface hydrophobicity, protease activity, and lipase activity [69]. Furthermore, palmitic acid has been observed to induce apoptosis and oxidative stress in *C. tropicalis* cells. This suggests that palmitic acid can trigger programmed cell death and generate an imbalance in the cellular redox status, leading to detrimental effects on the viability and survival of *C. tropicalis* [69]. Linoleic acid and linolenic acid can inhibit the biofilm formation and virulence of some bacteria, such as *Helicobacter pylori* and *P. aeruginosa*, by disrupting their membrane and fatty acid synthesis [53,57]. However, these fatty acids may not be effective against all types of bacteria and biofilms, and may require different concentrations or formulations to exert their antibiofilm activity. Similarly, phytol has been found to inhibit biofilm formation and quorum sensing in *Serratia marcescens*, another Gram-negative bacterium known to cause opportunistic infections. Phytol’s effects on *S. marcescens* involve impacting the bacterial cell membrane and modulating gene expression [61].

The combination of antimicrobial and antibiofilm compounds from the AAE and PME was shown to enhance their bioactivity and potency against multidrug-resistant (MDR) and pandrug-resistant (PDR) bacteria, specifically the potent *Pseudomonas aeruginosa* strain P17. This strain exhibited resistance to all tested antibiotics, including TPZ, CN, FOX, CFS, LE, NOR, AZM, STX, PI, TOB, MEM, AK, IPM, and CIP. However, mixing the AAE and PME extracts significantly increased the antimicrobial and antibiofilm activities, providing a more effective response against *P. aeruginosa* strains. This highlighted the potential of combining these extracts to combat drug-resistant bacteria and their biofilm formation. Further research and exploration are needed to fully understand the mechanisms behind this enhanced activity and to develop novel strategies for addressing the challenge of MDR and PDR bacterial infections.

Our analysis suggested optimal property ranges for both algal extracts, making them promising candidates for future antimicrobial agent design. These ranges included a molecular weight (MW) between 200 and 600, hydrogen bond acceptors ranging from 0 to 4, hydrogen bond donors between 0 and 5, a total polar surface area (TPSA) of less than 60 Å^2^ (except for bis (3-bromo-4,5-dihydroxybenzyl) ether), and a molar refractivity (MR) ranging from 40 to 130 m^3^/mol. These characteristics indicated their potential suitability for the development of antimicrobial agents. Additionally, the logarithm of the partition coefficient (M log P) fell between one and five, highlighting their lipophilicity. Notably, phytol in the AAE exhibited a high M log P value of 5.91, indicating a strong affinity for lipid-rich environments and nonpolar solvents. The hydrogen bond donors and acceptors fell within acceptable limits, facilitating interactions and binding with macromolecular targets [70]. This suggests that these compounds have the capacity to form stable complexes and exhibit desired biological activities through favorable hydrogen bonding interactions with target molecules.

The TPSA, or the total polar surface area, is a valuable parameter for predicting drug transport properties, particularly intestinal absorption. Typically, a TPSA value below 140 Å^2^ is indicative of favorable intestinal absorption. A TPSA value below 60 Å^2^ suggests the potential for brain penetration. Analyzing the predicted TPSA values of the studied compounds indicated that they possessed favorable intestinal absorption characteristics, making them potentially efficient candidates for absorption in the intestines.

In general, the TPSA values falling between 60 Å^2^ and 140 Å^2^ suggested limited permeability through the blood–brain barrier (BBB). Among the compounds analyzed, bis (3-bromo-4,5-dihydroxybenzyl) ether of the PME exhibited a TPSA value of 90.15 Å^2^. This suggested that this specific compound may have slightly reduced permeability across the blood–brain barrier compared to others in the study.

Molar refractivity, which measures the total polarizability of a substance, provides insights into molecular interactions and compound properties. It can estimate the electronic polarizability of single ions in solution and assess a compound’s behavior in solution and its potential for interactions with biological systems. For optimal oral bioavailability and absorption, the molar refractivity value typically needs to fall within the range of 40 to 130 m^3^/mol. Most of the compounds analyzed in this study exhibited molar refractivity values within this range, indicating favorable physicochemical properties that promote good intestinal absorption and oral bioavailability.

The favorable molar refractivity values suggested that these compounds have the potential for efficient absorption in the gastrointestinal tract and systemic distribution. Skin permeability, as indicated by Kp values between −8.0 and −1.0, implies good potential for skin penetration, which is relevant for topical applications and dermal drug delivery.

Cytochrome P450 enzymes (CYPs) are pivotal in metabolizing both endogenous and xenobiotic compounds within the body. Among the approximately 50 isoforms of CYP enzymes, isoforms 1A2, 2C9, 2C19, 2D6, and 3A4 account for over 90% of oxidative metabolic activities. Assessing potential compounds for inhibitory activity on specific CYP isoforms is crucial in drug development, as inhibition can disrupt the metabolism of medications.

Pattern recognition algorithms have been employed to identify potentially problematic fragments within compounds. PAINS (pan assay interference compounds) are compounds that contain substructures known to yield strong responses in various tests, regardless of the specific protein target. Fortunately, the compounds identified in this study lacked such warning substructures within their chemical structures.

Lead-likeness, a concept focusing on a compound’s drug-likeness and suitability for optimization, considers various properties. It aids in determining a compound’s potential as a lead candidate.

The synthetic accessibility score is a key factor when evaluating potential compounds. It measures the ease or difficulty of synthesizing a compound, with scores ranging from 1 (extremely easy) to 10 (very tough). In this study, most compounds had scores below five, indicating relatively straightforward synthetic accessibility. Notably, phytol of the AAE scored the highest at 4.3, while 3,4-dihydroxybenzaldehyde in the PME had the lowest score of 3.

These assessments collectively contributed valuable insights into the potential of these compounds for pharmaceutical and biomedical applications, guiding the selection of promising candidates for further research and potential therapeutic development. An important path to explore for developing drugs against microbial infections is the type II fatty acid biosynthesis pathway. This pathway involves the action of enzymes such as β-hydroxyacyl-(acyl carrier protein) (ACP) dehydratase (FabZ) during the fatty acid elongation cycle. FabZ plays a role in metabolizing various types of fatty acids in bacteria and parasites. This makes FabZ a potential drug target in different pathogens, including *Pseudomonas aeruginosa* [71], *Helicobacter pylori* [72]*, E. coli* [73], and *Plasmodium falciparum* [74]. FabZ inhibition has shown promise in tackling a wide range of bacteria, making it an attractive target for developing broad-spectrum antimicrobial and antiparasitic drugs.

The docking analysis revealed that several bioactive compounds from both the AAE and PME interacted with the FabZ protein in *Pseudomonas aeruginosa*, indicating their potential as inhibitors. Compounds with more negative free energy values exhibited stronger binding. Notably, “Bis (3-bromo-4,5-dihydroxybenzyl) ether,” “3-Bromo-4,5-dihydroxybenzaldehyde,” and “11,14-Eicosadienoic acid, methyl ester” demonstrated favorable binding affinities and lower inhibition constants, suggesting potent inhibitory effects. Combining these extracts could enhance their activity synergistically. These compounds exhibited strong interactions with specific amino acids in the FabZ protein. This binding suggested their potential to inhibit the enzyme, potentially limiting pathogen growth. Given that FabZ is present in various human pathogens, these findings hint at the potential of these compounds as broad-spectrum antibiotic candidates.

## 4. Materials and Methods

### 4.1. Materials

All chemicals used were of analytical ranking and were bought from Sangon Biotech Co., Ltd. (Chedun, Shanghai, China). The ingredients for the bacteria medium and antibiotics were purchased from Sigma-Aldrich (Poole, UK). Zarrouk’s medium ingredients for *Arthrospira platensis* were purchased from Sinopharm Chemical Reagent Co., Ltd. (Huangpu, Shanghai, China).

### 4.2. Algal Collection

In this study, two algae belonging to different phyla were used: *Arthrospira platensis* (cyanobacteria) and *P. scopulorum* Harvey (Rhodophyta). *A. platensis* was kindly provided by the phycology laboratory in the Department of Botany, Faculty of Science, Tanta University, Egypt. The cultivation of *A. platensis* involved using Zarrouk’s medium as described by Aiba [75]. The culture was maintained under controlled conditions, with a light intensity of 45 µ mole photon m^−2^ s^−1^ and a temperature of 30 ± 2 °C. The culture received sterilized ambient air by being passed through bacterial air filters with a pore diameter of 0.45 µ. The biomass of *A. platensis* was acquired at the end of the exponential growth phase (16th day) through centrifugation (TDL-8 M, Luxiangyi, Hunan, China) at 4000 rpm for 20 min. The pellet cells were then rinsed and washed three times with sterilized dist. H_2_O and dried in an oven at 45 °C.

*P. scopulorum*, a member of Rhodophyta, was collected from the coastal area of Marsa Matruh, Egypt (31_24058.900N, 27_00026.200E). The collected algae underwent a series of steps, including cleaning, identification, drying, and transformation into a fine powder, following the procedures described in a previous study conducted by researchers [46].

### 4.3. Preparation of Organic Algal Extracts

To prepare the organic algal extracts, 10 g of the *A. platensis* and *P. scopulorum* powder was separately mixed with 150 mL of acetone and methanol, respectively. The mixtures were soaked for 72 h at room temperature (25 ± 2 °C) in a shaking incubator. Afterwards, the extracts were filtered (Whatman filter paper no 1) and concentrated using a rotary evaporator at 35 °C (Savant SpeedVac SVC100, Thermo Fisher Scientific, Waltham, MA, USA). The resulting crude extracts were then suspended in dimethyl sulfoxide (DMSO, 0.01%) to achieve a final concentration of 2000 µg/mL and stored in a refrigerator at 4 °C until further use.

### 4.4. Bacterial Sample Collection

The study took place in a fish farm with limited production capacity located in the Abu-Arab district, Riyadh, Kufr El-Sheikh Governorate. From May 2021 to January 2023, a total of 61 water samples were collected from various locations within the farm. The samples were collected using sterile brown bottles, transported to the laboratory, and promptly stored at 4 °C. A bacteriological analysis was conducted within 6 h of sample collection.

### 4.5. Quantification and Identification of Pseudomonas aeruginosa

The collected water samples were diluted with 0.85% (g/L) saline solution and plated on various agar plates, including nutrient agar, MacConkey agar, mannitol salt agar, and blood agar. Bacterial growth was observed and subculturing was performed to purify the bacterial isolates. In total, 104 bacterial isolates were obtained; then, Gram staining was performed. Gram-negative isolates were selected and further subcultured on MacConkey agar; only nonlactose fermenting isolates were subcultured on cetrimide agar, which is considered to be a selective medium, as cetrimide inhibits bacterial growth, except for *P. aeruginosa,* and enhances fluorescein and pyocyanin pigment production, following the method described by Holt et al. The identification process involved evaluating the colony morphology, cultural characteristics, and biochemical tests, including the fermentation of glucose, lactose, sucrose, gelatinase, oxidase, and catalase, commonly used in microbial identification [76].

### 4.6. Antibiotic Susceptibility Test (Disc Diffusion Method)

To evaluate the susceptibility of the *P. aeruginosa* isolates (n = 23) to commonly used antibiotics in the aquaculture sector in Egypt, the disc diffusion technique [77] was employed. The antibiotic discs were obtained from Oxoid Ltd. Basing stock, UK, at concentrations as described in Table 1. The selected antibiotics included cephalexin (CN), cefoxitin (Fox), cefsulodin (CFS), tobramycin (TOB), amikacin (AK), ciprofloxacin (CIP), norfloxacine (NOR), levofloxacin (LE), meropenem (MEM), imipenem (IPM), azithromycin (AZM), piperacillin–tazobactam (TPZ), sulfonamides–trimethoprim (STX), and pipemidic acid (PI), as recommended by the National Antimicrobial Resistance Monitoring System records. Muller Hinton agar plates (Oxoid, UK) were utilized for the antibiotic susceptibility testing, and the plates were incubated at 37 °C for 18 h. The diameter of the growth inhibition zone was measured to determine the sensitivity of the isolates. The results were interpreted and classified as sensitive (S), intermediate (I), or resistant (R), according to the guidelines provided by the European Committee on Antimicrobial Susceptibility Testing (EUCAST) [78] clinical breakpoint. Intermediate susceptibility and sensitive isolates were labelled as nonresistant. The term multidrug-resistant (MDR) refers to a bacterium that is resistant to at least one antimicrobial agent in three or more categories [79], whereas pandrug-resistance (PDR) refers to bacteria that are resistant to all tested antimicrobial agents in all classes [80].

### 4.7. Quantitative Assessment of Biofilm Formation

A modified colorimetric microtiter plate assay, based on the method described by Stepanović [81], was used to quantify biofilm formation in the 23 *P. aeruginosa* isolates. The isolates were cultured in 5 mL of trypticase soy broth (TSB) and incubated at 37 °C for 24 h. After incubation, the cultures were diluted in TSB to reach a turbidity equivalent to the 0.5 McFarland standard. In a flat-bottomed polystyrene 96-well microtiter plate, each well containing 180 μL of TSB and 20 μL of the diluted bacterial suspensions was inoculated. The plate was then incubated at 37 °C for 24 h. Following incubation, the wells were washed three times with sterile phosphate-buffered saline (PBS, pH 7.3) to remove nonadherent cells. The adherent biofilms were fixed by adding 96% methanol to each well and incubating for 15 min. The methanol was discarded, and the plate was air-dried for 45 min.

To stain the biofilms, 200 μL of 0.1% crystal violet solution was added to each well and incubated at room temperature for 30 min. Unbound stain was removed by washing the plate with sterile deionized water and allowing it to dry. The bound dye was solubilized by adding 200 μL of 33% glacial acetic acid to each well. The optical density (OD) of the solubilized dye was measured at 620 nm using a microtiter plate reader. The experiment was performed in triplicate, and a cut-off value (ODc) was determined. The ODc was defined as being three times the standard deviation above the mean OD of the negative control. Biofilm formation was classified into four categories, based on the OD values: if OD < ODc, the biofilm formation was considered negative; if ODc < OD < 2xODc, the biofilm was categorized weak; if 2xODc < OD < 4xODc, the biofilm was classified moderate; and if OD > 4xODc, the biofilm formation was deemed strong [81].

### 4.8. Detection of Specific Biofilm Genes in Selected Bacterial Isolates

For the evaluation of the three biofilm-encoding genes (algD, pelf, and pslD) in *P. aeruginosa*, the polymerase chain reaction (PCR) method was employed. DNA extraction from the samples was conducted using the GeneJET Genomic DNA Purification Kit from Thermo Fisher Scientific, USA, following the manufacturer’s protocol. Specific primers for the amplification of the target genes were designed using Primer3 software, based on the nucleotide sequences obtained from GenBank (Table S4). The PCR reaction was carried out in a total volume of 25 µL, consisting of 12.5 µL of Taq Master Mix, 0.3 µM of each forward and reverse primer, 100 ng of template DNA, and nuclease-free water to complete the volume.

The PCR amplification conditions included an initial denaturation step at 95 °C for 5 min, followed by 30 cycles of denaturation at 94 °C for 30 s, annealing at 60 °C for 40 s, and extension at 72 °C for 40 s. A final elongation step was performed at 72 °C for 5 min. After the PCR amplification, the products were detected using gel electrophoresis on a 1.5% agarose gel at 80 volts for 45 min. The gel was then stained with Midori green and visualized, imaged, and analyzed using a gel documentation system (Analytic Jena, Biometra model, Biodoc analyzer, Jena, Germany).

### 4.9. Antibacterial Efficacy of Algal Extracts: Individual and Combination Assessments

In this study, the antimicrobial activity of the acetone and methanol extracts from each algal species, as well as their combination, was evaluated. The antibiotics tobramycin (TOB) and piperacillin–tazobactam (TPZ) were used as positive controls, and a disc impregnated with dimethyl-sulfoxide (DMSO, 0.01%) served as the negative control. These antibiotics were used to confirm the resistance of the tested isolates to them and demonstrate the effectiveness of the algal extracts in overcoming this resistance. The antimicrobial activity of the algal extracts and antibiotics was tested against the selected strong biofilm strains using the disc diffusion method, following the protocol described by El-Sayed [38]. All experiments were conducted in triplicate to ensure the accuracy and reliability of the results.

### 4.10. Determination of the Minimum Inhibitory Concentration (MIC) Using Broth Microdilution Technique

The minimum inhibitory concentration (MIC) assay of the algal extracts followed the resazurin-based microtiter dilution technique, as outlined by Elshikh et al. [82]. The bacterial cultures were initially grown overnight in Luria broth and then diluted to a concentration of 10^6^ colony-forming units per milliliter (CFU/mL) using a sterile medium.

To conduct the assay, two-fold serial dilutions of algal extract were prepared, spanning concentrations from 2000 to 31.25 μg/mL. In a 96-well microtiter plate, we added 100 μL of the test organism (10^6^ CFU/mL) and 100 μL of the respective algal extract dilution to each well. The plate also included a positive control with bacteria and media, as well as a negative control with media only. Subsequently, the plates were incubated at 37 °C for 24 h.

After incubation, we introduced 40 µL of membrane-filtered resazurin dye (0.015% *w/v* in distilled water) to each well. Following an additional 2 h of incubation at 37 °C, we assessed the wells for color changes. The transformation of purple resazurin into fluorescent pink indicated actively metabolizing cells, while a dark blue color signified the complete inhibition of bacterial growth. The MIC was determined as the lowest concentration of algal extract at which the resazurin remained blue, indicating the full suppression of visible microbial growth.

### 4.11. Determination of Antibiofilm Activity and Biofilm Inhibitory Percentage for the Algal Extracts

The antibiofilm activity of the algal extracts was assessed for the selected MDR *P. aeruginosa* strains using a sterile flat-bottomed transparent 96-well polystyrene (PS) microtiter plate with a lid. Each well contained trypticase soy broth (TSB) and pathogens [83,84]. The algal extracts were added and mixed, and the minimum inhibitory concentration (MIC) against biofilm formation was determined through a dilution series. Bacteria-free mixtures were used to account for the extract components’ absorbance. The plate was incubated and nonadherent cells were removed through washing. Sessile bacteria were fixed, stained, and washed. The absorbance at 620 nm was measured. The percentage of the biofilm formation inhibition was calculated using the following equation:

$$\%\;Biofilm\;inhibition=\frac{\left(OD620\;of\;cells\;treated\;with\;algal\;extracts\right)}{\left(OD620of\;the\;nontreated\;control\right)}\times 100$$

The experiments were performed in triplicate and the mean standard deviation was determined.

### 4.12. Molecular Identification of the Selected MDR P. aeruginosa Strain

The molecular identification of the selected isolate was conducted according to the method of Ponnanikajamideen et al. [41]. The bacterial isolate was cultured in a nutrient broth medium at 28 °C for 48 h. DNA was extracted using a Patho Gene-spin DNA/RNA extraction kit (Korean company Intron Biotechnology). The PCR was performed using 16s rRNA universal primers (27F and 1492R) [85], and the resulting PCR products were confirmed using agarose gel electrophoresis. The purified amplicons were sequenced in both forward and reverse directions. The obtained sequences were analyzed using BLAST on the NCBI website, and the phylogenetic analysis was conducted using MegAlign software version 5.05.

### 4.13. Scanning Electron Microscopy (SEM)

For the SEM analysis, the method of Zammuto et al. [42] was conducted. In brief, individual PS coupons with specific dimensions were placed horizontally in 12-well polystyrene microtiter plates for the SEM analysis. Each well received 300 µL of TSB containing antimicrobial compounds at a ½ MIC concentration. A negative control with only TSB was included. The bacterial suspension (106 CFU/mL) was added to the wells and the plates were incubated statically at 30 °C for 24 h. After the incubation, the chips were cleaned with sterile PBS, treated with glutaraldehyde, and dehydrated with alcohol. The samples were then mounted on aluminum stubs and examined using SEM (JSM-6510 LV, IEOL, Japan) after critical-point drying and gold coating in an SPI- Module (Sputter Carbon/Gold COATER).

### 4.14. Determination of Gene Expression with Quantitative Real-Time PCR

To assess the three biofilm genes (algD, pelf, and pslD) in *P. aeruginosa*, we employed the PCR. DNA extraction used the GeneJET Genomic DNA Purification Kit from Thermo Fisher Scientific, USA, per the manufacturer’s protocol. Gene-specific primers were designed using Primer3 software, based on GenBank’s nucleotide sequences (Table S4). The PCR mix contained 12.5 µL of Taq Master Mix, 0.3 µM of each forward and reverse primer, 100 ng of template DNA, and nuclease-free water in a 25 µL total volume.

The PCR conditions were: initial denaturation at 95 °C for 5 min, followed by 30 cycles of denaturation at 94 °C for 30 s, annealing at 60 °C for 40 s, and extension at 72 °C for 40 s, with a final elongation step at 72 °C for 5 min. Products were detected with 1.5% agarose gel electrophoresis at 80 volts for 45 min, stained with Midori green, and visualized using a gel documentation system (Analytic Jena, Biometra model, Biodoc analyzer, Germany).

A quantitative RT-PCR was used to assess the impact of the antimicrobial compounds on gene expression related to biofilm formation [86,87]. Bacteria were cultured in the presence or absence of antimicrobial compounds (½ × MIC) for 72 h. Total RNA was extracted using the TRIzol reagent from the biofilm-forming bacteria and the extracted RNA was reverse-transcribed into cDNA using the QuantiTect Reverse Transcription Kit and a random primer hexamer in a two-step RT-PCR. The 2-ΔΔCt method was utilized to analyze the relative gene expression based on the melting curve. The 16sRNA was considered as the housekeeping gene to determine the expression level. The fold change in the gene expression was determined relative to a reference gene (16s rRNA) and validated using data analysis tools.

### 4.15. GC–MS Characterization

The analysis of the phycocompounds in each extract was conducted using a gas chromatography–mass spectrometer (GC–MS) system (Agilent 6890 GC coupled to an Agilent 5975 quadrupole mass detector, Palo Alto, CA, USA). The GC–MS instrument was equipped with a fused capillary column (HP-5MS) containing 5% phenyl methyl siloxane and having a film thickness of 30 m × 0.25 mm × 0.25 μm (J&W Scientific, Folsom, CA, USA).

For the GC analysis, the temperature of the chromatography oven was programmed to increase from 60 to 250 °C at a rate of 2 °C per minute. The carrier gas used was helium with a constant flow rate of 0.9 mL per min. The injection volume was set to 1 μL with a split ratio of 10:1.

To identify unknown compounds, they were compared with standard data available in the NIST library version 2005. The identified compounds were subjected to further analysis for their biological activity, toxicity prediction, and molecular docking interactions using in silico methods.

### 4.16. Evaluation of Pharmacokinetics and Toxicity

The simplified molecular input line entry system (SMILES) notations obtained from the GC/MS results were collected from the PubChem database (https://pubchem.ncbi.nlm.nih.gov/) (accessed on 15 July 2023) [88]. These structures were used for predicting the biological effects, toxicity, multitarget ligands, and potential drug targets of the compounds. This information could be valuable for drug repurposing or predicting off-target effects.

The collected SMILES notations were also utilized for the ADME (absorption, distribution, metabolism, and excretion) analysis. The SwissADME program, available online at http://www.swissadme.ch (accessed on 15 July 2023), was employed to assess the drug-likeness and pharmacokinetic properties of the identified compounds from the algal extract. Lipinski’s rule of 5, a set of criteria for drug-likeness, was applied to evaluate the compounds [70,89].

Various pharmacokinetic properties were examined, including the molar refractivity (MR), skin permeability (log Kp), permeability of glycoprotein substrate (Pgp), gastrointestinal absorption, blood–brain barrier (BBB) penetration, and cytochrome P450 (CYP450) enzyme inhibition [90,91]. These assessments provided valuable insights into the potential pharmacological properties and suitability of the identified compounds for drug development.

The toxicity profiles of the identified phycocompounds were predicted using the ProTox-II webserver (https://tox-new.charite.de/protox_II, accessed on 15 July 2023) [91,92].

### 4.17. Molecular Docking Studies

The atomic coordinates of *Pseudomonas aeruginosa*’s FabZ enzyme (3R)-hydroxyacyl-acyl carrier protein dehydratase (PDB ID: 1U1Z) were retrieved from the Protein Data Bank. Subsequently, the ligand file was created using Discovery Studio, with force field parameters assigned for the ligand’s ionization at pH 7, followed by the generation of the ligand’s 3D structure. The molecular docking analysis was conducted using the docking server available at https://www.dockingserver.com/web (accessed on 15 July 2023).

### 4.18. Statistical Analysis

The statistical analysis was conducted using SPSS software (version 23, IBM Corp., Armonk, NY, USA). All experiments were performed with three independent replicates. The analysis of variance (ANOVA) was employed to determine the statistical significance of the differences among the experimental groups. A post hoc analysis was carried out using the Tukey test to compare the means between the groups. Statistical significance was set at *p* < 0.05.

## 5. Conclusions

Two specific algal extracts, the *Arthrospira platensis* acetone extract (AAE) and *Polysiphonia scopulorum* methanol extract (PME), as well as their combination (COE), exhibited promising antibacterial and antibiofilm activities. The COE showed superior effects compared to individual extracts, resulting in a significant downregulation of specific genes associated with *P. aeruginosa* pathogenicity. The observed effects of the algal extracts were attributed to their fatty acid content, along with specific compounds, such as phytol and bromophenol. The analysis using the SwissDock Web server indicated favorable physicochemical properties of the algal extracts for potential use as antimicrobial agents. The presence of these phytochemical compounds with promising drug-like and pharmacokinetic properties further supports their potential application in clinical settings. The ability of the combined algal extracts to exhibit enhanced antibacterial and antibiofilm efficacy against these resistant strains is encouraging and suggests the potential for developing alternative treatment options. The synergistic action of the combined algal extracts highlights their potential in addressing drug-resistant infections and calls for further investigation.

## Figures and Tables

**Table 1:** Antimicrobial susceptibility patterns of the 23 *Pseudomonas aeruginosa* isolates isolated from water from a fish farm.

Antibiotic Class	Antimicrobial Agent	Disc Potency (µg/Disc)	Resistant Strains		Susceptible Strains	
			No.	%	No.	%
Cephalosporin	Cephalexin (CN)	3	20	86.9%	3	13.1%
	Cefoxitin (Fox)	30	20	86.9%	3	13.1%
	Cefsulodin (CFS)	30	20	86.9%	3	13.1%
Aminoglycosides	Tobramycin (TOB)	10	7	30.4%	16	69.5%
	Amikacin (AK)	30	3	13.1%	20	86.9%
Fluoroquinolones	Ciprofloxacin (CIP)	5	1	4.34%	22	95.6%
	Norfloxacine (NOR)	10	10	43.4%	13	56.5%
	Levofloxacin (LE)	5	11	47.8%	12	52.1%
Carbapenem	Meropenem (MEM)	10	4	17.3%	19	82.6%
	Imipenem (IPM)	10	1	4.34%	22	95.6%
Macrolide	Azithromycin (AZM)	30	15	65.2%	8	34.7%
Penicillin	Piperacillin–tazobactam (TPZ)	75/10	20	86.9%	3	13.1%
Sulfonamides–trimethoprim	Sulfonamides–trimethoprim (STX)	25	12	52.1%	11	47.8%
Quinolones	Pipemidic acid (PI)	20	15	65.2%	11	34.7%

**Table 2:** Antimicrobial resistance patterns of MDR *P. aeruginosa* isolates.

Pattern Code		Resistance Pattern Profile	No. of MDR Isolates (23)	Isolate Code Exhibited by Pattern
I	a	TPZ, CN, FOX, CFS, and AZM	3	3, 11, 16
	b	TPZ, CN, FOX, CFS, and PI	3	10, 21, 22
	c	TPZ, CN, FOX, CFS, and LE	2	4, 5
II	a	TPZ, CN, FOX, CFS, LE, AZM, STX, and PI	2	6, 9
	b	TPZ, CN, FOX, CFS, NOR, AZM, STX, and PI	3	15, 20, 23
III		TPZ, CN, FOX, CFS, LE, NOR, AZM, STX, PI, and TOB	4	12,14, 18, 19
IV		TPZ, CN, FOX, CFS, LE, NOR, AZM, STX, PI, TOB, MEM, and AK	2	1, 13
V		TPZ, CN, FOX, CFS LE, NOR, AZM, STX, PI, TOB, MEM, AK, IPM, and CIP	1	17

**Table 3:** Biofilm-producing abilities of the 23 *P. aeruginosa* isolates.

Bacterial Strain Code	Biofilm Producing Ability (BPA)		Classification according to Antibiotic Resistance
	OD ± SD	Strength	
P1	0.58 ± 0.02 ^a^	Strong	MDR
P2	0.22 ± 0.04 ^h^	Weak	Sensitive
P3	0.14 ± 0.06 ^k^	Weak	MDR
P4	0.22 ± 0.07 ^h^	Weak	MDR
P5	0.15 ± 0.04 ^k^	Weak	MDR
P6	0.32 ± 0.02 ^f^	Moderate	MDR
P7	0.22 ± 0.03 ^h^	Weak	Sensitive
P8	0.22 ± 0.05 ^h^	Weak	Sensitive
P9	0.26 ± 0.04 ^g^	Moderate	MDR
P10	0.19 ± 0.05 ^i^	Weak	MDR
P11	0.21 ± 0.05 ^h^	Weak	MDR
P12	0.37 ± 0.05 ^d^	Moderate	MDR
P13	0.54 ± 0.02 ^b^	Strong	MDR
P14	0.32 ± 0.05 ^f^	Moderate	MDR
P15	0.32 ± 0.01 ^f^	Moderate	MDR
P16	0.15 ± 0.06 ^k^	Weak	MDR
P17	0.60 ± 0.04 ^a^	Strong	PDR
P18	0.37 ± 0.04 ^d^	Moderate	MDR
P19	0.25 ± 0.04 ^g^	Moderate	MDR
P20	0.34 ± 0.05 ^e^	Moderate	MDR
P21	0.18± 0.05 ^j^	Weak	MDR
P22	0.21 ± 0.05 ^h^	Weak	MDR
P23	0.43 ± 0.05 ^c^	Moderate	MDR
F-value	3114.33	*p*-value	<0.001

Data illustrate the average ± standard deviation of three replicates. Distinct letters in the given column indicate significant differences (*p* < 0.05), as determined through post hoc Duncan testing.

**Table 4:** Average inhibition zone diameters produced with algal extracts and two commercial antibiotics against tested *P. aeruginosa* strains.

Microbial Strain	Mean of Diameter of Inhibition Zones (mm)						
	*A. platensis*		*P. scopulorum*		AAE + PME (COE)	TPZ	TOB
	**Methanol**	**Acetone**	**Methanol**	**Acetone**			
P1	6.1± 0.02 ^a^	11.66 ± 0.94 ^a^	16 ± 0.81 ^a^	6.8 ± 0.21 ^c^	27.66 ± 0.94 ^b^	0.0 ± 0.0	0.0 ± 0.0
P13	6.8 ± 0.13 ^b^	10.66 ± 0.47 ^c^	25.66 ± 0.94 ^b^	7.3 ± 0.11 ^b^	36 ± 0.81 ^a^	0.0 ± 0.0	0.0 ± 0.0
P17	6.5 ± 0.05 ^c^	11 ± 0.47 ^b^	15.33 ± 0.47 ^a^	7.5±0.23 ^a^	18.33 ± 0.47 ^c^	0.0 ± 0.0	0.0 ± 0.0
F-value	9676.42	811.71	55.84	5207.11	245,428.04		
P-value	<0.001	<0.001	<0.001	<0.001	<0.001		

Results expressed as mean ± standard deviation of three replicates; AAE—*A. platensis* acetone extract; PME—*P. scopulorum* methanol extract; COE—combined extract. Distinct letters in the given column indicate significant differences (*p* < 0.05), as determined through post hoc Duncan testing.

**Table 5:** Chemical constituents of *A. platensis* acetone extract.

No.	Compound Name	Peak Area %	Molecular Formula
1	3,7,11,15-Tetramethyl-2-hexadecen-1-ol (phytol)	36.6	C_20_H_40_O
2	n-Hexadecanoic acid (palmitic acid)	21.01	C_16_H_32_O_2_
3	11,14-Eicosadienoic acid, methyl ester	12.68	C_21_H_38_O_2_
4	Trans-13-octadecenoic acid (elaidic acid)	7.67	C_18_H_34_O_2_
5	Linoleic acid ethyl ester	7.42	C_18_H_32_O_2_
6	9,12,15-Octadecatrienoic acid	3.44	C_18_H_30_O_2_
7	8,11,14-Eicosatrienoic acid, (Z,Z,Z)-	3.11	C_20_H_34_O_2_
8	Cholesta-8,24-dien-3-ol, 4-methyl-, (3á,4à)-	2.89	C_28_H_46_O
9	Hexadecenoic acid, Z-11-	1.72	C_16_H_30_O_2_
10	Octadecanoic acid	1.08	C_18_H_36_O_2_
11	Decane, 2,9-dimethyl	1.03	C_12_H_26_
12	Tridecane	0.88	C_13_H_28_
13	Hexadecane	0.47	C_16_H_34_

**Table 6:** Chemical constituents of *P. scopulorum* methanol extract.

No.	Compound Name	Peak Area %	Molecular Formula
1	n-Hexadecanoic acid methyl ester	22.75	C_16_H_32_O_2_
2	Bis (3-bromo-4,5-dihydroxybenzyl) ether	13.25	C_14_H_12_Br_2_O_5_
3	3-Bromo-4,5-dihydroxybenzaldehyde	10.76	C_7_H_5_BrO_3_
4	3,4-Dihydroxybenzaldehyde	9.71	C_7_H_6_O_3_
5	Linolenic acid methyl ester	8.7	C_18_H_30_O_4_
6	Eicosane	3.95	C_20_H_42_
7	Myristic acid methyl ester	3.63	C_14_H_28_O_2_
8	Linoleic acid	3.52	C_18_H_32_O_2_
9	Octadecanoic acid	3.24	C_18_H_36_O_2_
10	Hexadecane	3.11	C_16_H_34_
11	24-Methyl-cholesta-5,22Z-dien-3ß-ol	3.08	C_31_H_52_O
12	Octadecane	1.89	C_18_H_38_
13	Nonadecane	1.83	C_19_H_40_
14	Phytol	1.77	C_20_H_40_O
15	Dodecane, 2,6,10-trimethyl	1.71	C_15_H_32_
16	Cis-13-octadecenoic acid	1.19	C_18_H_34_O_2_
17	Phthalic acid,	1.11	C_8_H_6_O_4_
18	Fucosterol	1.09	C_2_9H_48_O
19	24-Ethyl-cholest-5-en-3ß-ol	0.71	C_2_9H_50_O

## Data Availability

Not applicable.

## Supplementary Materials

The following supporting information can be downloaded at: https://www.mdpi.com/article/10.3390/plants12183324/s1; Figure S1: Some morphological and biochemical characters of the isolated *P. aeruginosa*; Table S1: Physicochemical properties, water solubility and pharmacokinetic properties of A. platensis acetone extract by Swiss ADME; Table S2: Physicochemical properties, water solubility and pharmacokinetic properties of *P. scopulorum* methanol extract by Swiss ADME; Table S3: Molecular docking data presents results from docking simulations of various phycocompounds from the PME and AAE; Table S4: primer sequences used in this study.
