# Supplementary material for: Unveiling the Potential of Algal Extracts as Promising Antibacterial and Antibiofilm Agents against Multidrug-Resistant Pseudomonas aeruginosa: In Vitro and In Silico Studies including Molecular Docking
Source: Plants (Basel). 2023 Sep 20;12(18):3324. doi: 10.3390/plants12183324 (PMC10537748; doi:10.3390/plants12183324)
Supplement: Supplementary file 1 [file plants-12-03324-s001.zip › plants-2591457-supplementary.pdf]

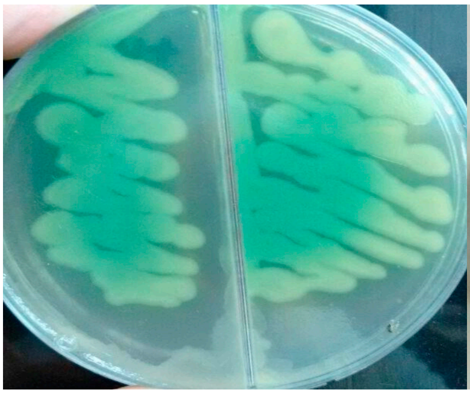

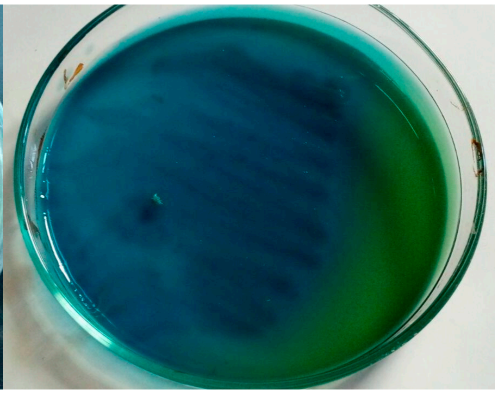

Pyocyanin pigment on cetrimide agar medium

Positive citrate test

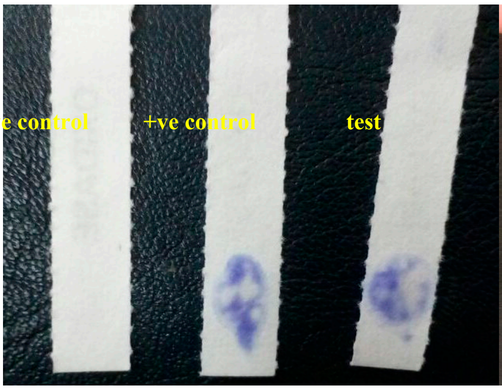

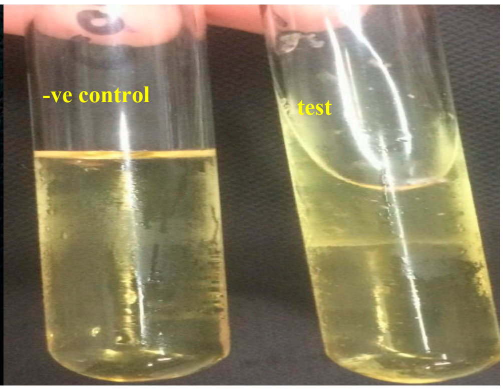

Positive oxidase test

Positive gelatinase test

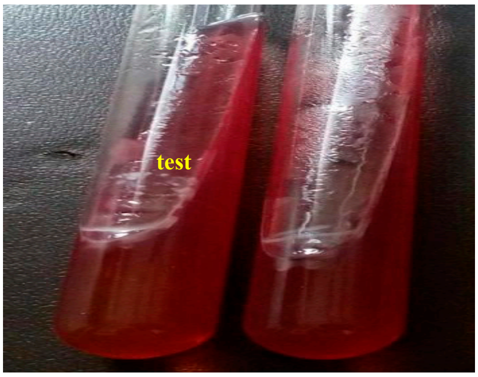

Triple sugar iron agar test tube (-ve)

Figure S1: Some morphological and biochemical characters of the isolated *P. aeruginosa*

Table S1. Physicochemical properties, water solubility and pharmacokinetic properties of *A. platensis* acetone extract by *Swiss ADME*.

**Dominate compounds**	**Linoleic acid ethyl ester**	**3,7,11,15-tetramethyl-2-hexadecen-1-ol**	***n*-hexadecanoic acid**	**11,14-eicosadienoic acid, methyl ester**	**Trans-13-octadecenoic acid**
**Physicochemical Properties**					
MW	312.44	256.42	338.61	366.53	296.49
#Heavy atoms	22	18	24	26	21
#H-bond acceptors	4	2	1	4	2
#H-bond donors	0	1	1	0	1
MlogP	3.55	4.19	5.91	4.36	4.8
MR	90.74	80.8	112.84	109.49	94.74
**Solubility**					
Esol log s	-3.69	-5.98	-5.02	-5.69	-5.41
Esol class	Soluble	Moderately soluble	Moderately soluble	Moderately soluble	Moderately soluble
Silicos-it logsw	-4.96	-5.51	-5.31	-6.17	-5.39
Silicos-it class	Moderately soluble	Moderately soluble	Moderately soluble	Poorly soluble	Moderately soluble
**Pharmaco-kinetics**					
Cyp1a2 inhibitor	No	No	Yes	Yes	Yes
Cyp2c19 inhibitor	No	No	No	No	No
Cyp2c9 inhibitor	No	Yes	Yes	Yes	Yes
Cyp2d6 inhibitor	No	No	No	No	No
Cyp3a4 inhibitor	Yes	No	No	No	No
Log kp (cm/s)	-4.85	-2.29	-2.77	-2.66	-2.6
**Likeness drug**					
Lipinski #violations	0	1	1	1	1
Bioavailability score	0.55	0.55	0.85	0.55	0.85
PAINS #alerts	0	0	0	0	0
Synthetic accessibility	3.11	4.3	2.31	3.41	3.07
**Toxicity**					
Hepatotoxicity	inactive	inactive	inactive	inactive	inactive
cytotoxicity	inactive	inactive	inactive	inactive	inactive

Table S2. Physicochemical properties, water solubility and pharmacokinetic properties of *P. scopulorum* methanol extract by *Swiss ADME*.

**Dominate compounds**	**Linolenic acid methyl ester**	**n-hexadecanoic acid methyl ester**	**3-bromo-4,5-dihydroxybenzaldehyde**	**Bis (3-bromo-4,5-dihydroxybenzyl) ether**	**3,4-dihydroxybenzaldehyde**
**Physicochemical Properties**					
MW	278.43	240.38	217.02	420.05	138.12
#Heavy atoms	20	17	11	21	10
#H-bond acceptors	0	0	3	5	3
#H-bond donors	1	0	2	4	2
MlogP	4.38	3.84	0.94	2.21	0.18
MR	88.99	75.03	43.58	85.28	35.88
**Solubility**					
**Esol log s**	-4.78	-3.86	-2.83	-4.51	-1.76
**Esol class**	Moderately soluble	Soluble	Soluble	Moderately soluble	Very soluble
**Silicos-it logsw**	-3.96	-4.49	-3.46	-4.77	-1.17
**Silicos-it class**	Soluble	Moderately soluble	Soluble	Moderately soluble	Soluble
**Pharmaco-kinetics**					
**Cyp1a2 inhibitor**	Yes	Yes	Yes	Yes	No
**Cyp2c19 inhibitor**	No	No	Yes	No	No
**Cyp2c9 inhibitor**	No	No	Yes	Yes	Yes
**Cyp2d6 inhibitor**	No	No	Yes	Yes	No
**Cyp3a4 inhibitor**	No	No	Yes	Yes	No
**Log kp (cm/s)**	-4.02	-6.3	-6.66	-6.71	-3.04
**Likeness drug**					
**Lipinski #violations**	1	0	0	0	0
**Bioavailability score**	0.85	0.55	0.55	0.55	0.55
**PAINS #alerts**	0	0	1	0	1
**Synthetic accessibility**	3.03	2.71	1.49	2.30	1
**Toxicity**					
**Hepatotoxicity**	inactive	inactive	Inactive	inactive	inactive
**cytotoxicity**	inactive	inactive	inactive	inactive	inactive

Table S3: Molecular docking data presents results from docking simulations of various phycocompounds from PME and AAE

Extract	phycocompounds	**Est. Free Energy of Binding** (kcal/mol)	**Est. Inhibition Constant, Ki** (mM)	**vdW + Hbond + desolv Energy** (kcal/mol)	**Electrostatic Energy** (kcal/mol)	**Total Intermolec. Energy** (kcal/mol)
PME	Linolenic acid methyl ester	-2.11	28.56	-5.02	+0.14	-4.88
	3-Bromo-4,5-dihydroxybenzaldehyde	-4.54	0.47	-3.65	-0.41	-4.06
	Bis (3-bromo-4,5-dihydroxybenzyl) ether	-4.59	0.43	-4.46	-0.35	-4.80
	3,4-dihydroxybenzaldehyde	-3.72	1.89	-3.91	-0.10	-4.01
	n-Hexadecanoic acid methyl ester	-1.31	110.00	-5.32	-0.03	-5.34
AAE	Linolenic acid ethyl ester	-1.59	68.60	-5.19	-0.07	-5.26
	11,14-Eicosadienoic acid, methyl ester	-2.50	14.71	-6.57	-0.05	-6.63
	trans-13-Octadecenoic acid	-2.59	12.55	-5.23	-0.93	-6.15
	3,7,11,15-Tetramethyl-2-hexadecen-1-ol	-2.75	9.66	-5.90	-0.04	-5.93
	n-Hexadecanoic acid	-1.96	36.36	-6.09	+0.38	-5.71

Table S4. Primer sequences used in this study.

Genes	Sequences
algD	F-CTACATCGAGACCGTCTGCC<br>R-GCATCAACGAACCGAGCATC
pelF	F-GAGGTCAGCTACATCCGTCG<br>R-TCATGCAATCTCCGTGGCTT
pslD	F- TGTACACCGTGCTCAACGAC<br>R- CTTCCGGCCCGATCTTCATC
Reference gene *16S rRNA*	GCTGCCCTTTGTATTGTC<br>AGATGTTGGGTTAAGTCCC
